# In Ovo Injection of GABA Can Help Body Weight Gain at Hatch, Increase Chick Weight to Egg Weight Ratio, and Improve Broiler Heat Resistance

**DOI:** 10.3390/ani11051364

**Published:** 2021-05-11

**Authors:** Chris-Major Ncho, Akshat Goel, Chae-Mi Jeong, Mohamed Youssouf, Yang-Ho Choi

**Affiliations:** 1 Department of Animal Science, Gyeongsang National University, Jinju 52828, Korea; major159@gnu.ac.kr (C.-M.N.); genesakshat@gnu.ac.kr (A.G.); wjdcoa@gnu.ac.kr (C.-M.J.); youssoufzainou@yahoo.fr (M.Y.); 2 Division of Applied Life Sciences (BK21 Plus Program), Gyeongsang National University, Jinju 52828, Korea; 3 Institute of Agriculture and Life Sciences, Gyeongsang National University, Jinju 52828, Korea

**Keywords:** in ovo feeding, GABA, heat stress, broiler chicken, antioxidant status, fatty acid metabolism

## Abstract

**Simple Summary:**

Heat stress is a vital issue that causes severe losses to the poultry industry. A partly developed thermoregulatory mechanism during the embryonic phase is emphasized to manipulate embryos for achieving thermotolerance during rearing. The present study was conducted firstly to standardize the dosage for an in ovo manipulation, and the selective dose was used to evaluate its effects on early-age heat-stressed (HS) broilers. HS induces cholesterol while an antioxidant acts as a first line of defense under stress. However, 5% GABA supplementation had a higher hatchling weight and chick weight to egg weight ratio (CWEWR). We selected a 10% GABA dosage for HS studies due to its higher antioxidants and lower cholesterol values in hatchlings. In ovo, 10% GABA supplementation significantly increased total antioxidant capacity and reduced malondialdehyde levels, hepatic mRNA levels of HSP70, FAS, and L-FABP in broilers when subjected to HS (38 ± 1 °C; 3 h) at ten days of age. This indicates that an in ovo GABA injection improves CWEWR and antioxidant status at hatch, and creates thermotolerance by increasing antioxidant production and downregulating the expression of HSP70 and fatty acid metabolism genes in HS chicks.

**Abstract:**

The aim of this study was to explore the outcomes of an in ovo GABA injection in broilers challenged with HS. In Experiment 1, 210 Arbor Acres eggs were allocated to five treatments: no-injection, and in ovo injection of 0.6 mL of 0%, 5%, 10%, or 20% of GABA. Hatchling weight and CWEWR were significantly increased in the 5% GABA group. In ovo, injection of 10% GABA solution caused a significant decrease in plasma cholesterol and increased plasma total antioxidant capacity of hatchlings. Experiment 2 was conducted with 126 fertile Arbor Acres eggs distributed into one of two groups. At 17.5 days of incubation, one received no injection, and the other was fed 0.6 mL of 10% GABA. On day 10, one subgroup (4 replicates * 3 birds) from each treatment was submitted to HS (38 ± 1 °C for 3 h) while the other was kept at a thermoneutral temperature (29 ± 1 °C). An in ovo injection of GABA significantly increased total antioxidant capacity, but reduced malondialdehyde levels, hepatic mRNA levels of HSP70, FAS, and L-FABP with HS. In conclusion, an in ovo GABA injection improves CWEWR and antioxidant status at hatch, and enhances antioxidant status while downregulating the expression of HSP70 and fatty acid metabolism-related genes in young chicks under HS.

## 1. Introduction

As the global climate changes, environmental variations have become a striking challenge for animal production [1]. Especially in poultry farming, modern broiler breeds are extensively submitted to selection for increasing their growth performances under mild climates [2]. Moreover, the absence of sweat glands and the presence of feathers mean that chickens have a poor ability to regulate body temperature in high-temperature environments [3].

Heat stress (HS) can affect the performance of poultry at any stage of their life. Raising birds at high ambient temperatures has a number of drawbacks. Some of the deleterious effects of HS can be seen in growth performance, reproductive function, and immune response. HS is also one of the main causes of decreasing antioxidant capacity [4].

Multiple strategies have been considered to alleviate the harmful effects of HS in chickens. Recently, an in ovo injection was also included in mitigation solutions. An in ovo injection of L-leucine, for example, proved to be effective in affording thermotolerance to broilers under acute HS, mainly by altering amino acid metabolism [5]. An in ovo injection of galactooligosaccharides reduced the harmful effects of hyperthermia on feed efficiency in the finisher phase [6]. A better thermoregulatory mechanism under HS was reported in young chickens produced after an in ovo administration of high doses of L-leucine [7].

γ-aminobutyric acid (GABA) is a four-carbon non-protein amino acid primarily involved in inhibitory synaptic transmission [8]. GABA is involved in several regulatory functions such as memory, blood pressure, and respiration [9]. In chickens, GABA is used as a feed supplement for its ability to reduce the adverse effects of high environmental temperatures. Previous studies demonstrated its effectiveness for increasing feed intake, improving nutrient absorption, reducing oxidative stress in broilers [10,11,12], and decreasing mortality in laying hens [13] at elevated high temperatures.

Therefore, we hypothesized that an in ovo injection of GABA can effectively mitigate the detrimental effects of HS in broilers. In the current study, a dose-dependent study was first performed to determine an effective dose for an in ovo GABA supplementation at 17.5 days of embryonic life, and the dose selected was then used to test the effects in ten-day-old chicks under HS.

## 2. Materials and Methods

All the relevant procedures were approved by the Animal Care and Use Committee of Gyeongsang National University (GNU-200916-C0058). Two experiments were performed. The objective of Experiment 1 was to standardize the doses of an in ovo injection of GABA, and that of Experiment 2 was to evaluate HS responses in ten-day-old chicks after an in ovo injection of GABA at 10%.

### 2.1. Incubation and In-Ovo Procedures

#### 2.1.1. Experiment 1

Eggs laid from 40-week-old Arbor Acres breeder hens were obtained from a local breeder farm (Hapcheon, Korea). A total of 252 eggs were weighed (61.0 ± 0.2 g), labeled individually, and incubated in an incubator (Rcom Co., Ltd., Kimhae, Korea) with standard conditions (37.8 °C and 56% relative humidity) from embryonic days (EDs) 1 to 17, and then 36.8 °C and 70% from EDs 18 to 21. On ED 10, eggs were candled, and a total of 210 remained (61.3 ± 0.3 g) for the trial. Thereafter, the eggs were assigned to one of five treatment groups: 1) un-injected control (CON); 2) distilled water (DW)-injected control (DDW); 3) 5% GABA (G05); 4) 10% GABA (G10); and 5) 20% GABA (G20). DW was used as a diluent-injected control according to a previous study [14]. Within 3 h before the injection, GABA (#A2129 Sigma-Aldrich Inc., St. Louis, MO, USA) was dissolved in DW to obtain 0%, 5%, 10%, and 20% solutions for injection. The solutions were then stored at 30 °C until the injection was complete. The selected injection day was ED 17.5 because in ovo vaccination is usually executed between ED 17.5 to 19.25 [15], and the target of our injection was the amniotic sac of the embryo. Briefly, on ED 17.5, individual eggs received 0.6 mL of each solution at the blunt end using a 1 mL syringe with a 23-G and 1-inch needle. Therefore, the total amount of GABA injected into each egg was 0, 30, 60, and 120 mg for DDW, G05, G10, and G20 treatments, respectively. Before injection, the blunt end of each egg was disinfected with a 70% ethanol solution and a small hole was drilled using a dental drill (Saeshin, Daegu, Korea). After the injection, the egg hole made was sealed with surgical tape (3M Micropore, Saint Paul, Mn, USA) and placed back in its own incubator. In the case of CON, the eggs were taken out of the incubator and left for the same time without injection, and then returned to the incubator. Each treatment group consisted of 42 eggs (7 replicates of 6 eggs each). The eggs were turned every hour until ED 18.

#### 2.1.2. Experiment 2

The incubation procedures were exactly the same as described above, except for breeders’ age and the number of eggs used. A total of 150 eggs was obtained from 30-wk-old breeder hens. The in ovo injection procedure was similar to that in Experiment 1. At 17.5 days of incubation, there were two groups of eggs, with one group not injected (G0) and the other with 0.6 mL of 10% GABA (G10) dissolved in distilled water. After candling, 126 remaining eggs were assigned to one of the two groups composed of 63 eggs each. 

### 2.2. Feeding Experiment and HS Challenge

A total number of 56 chicks (28 for G0 and 28 for G10) were reared in battery cages with water and feed available ad libitum. On the tenth day, 48 chicks were selected based on their average body weight, sorted, and distributed into one of four treatment groups: (1) chicks, hatched from un-injected eggs (G0), and placed at a thermoneutral temperature (TN) (G0-TN); (2) chicks hatched from un-injected eggs (G0) but placed at a high temperature (HS) (G0-HS); (3) chicks, hatched from eggs injected with 10% GABA (G10), and placed at a TN (G10-TN); and (4) chicks, hatched from eggs injected with 10% GABA (G10), and placed under HS (G10-HS). Each treatment had 12 chicks kept in four cages with three chicks in each cage. Chicks were housed in metabolic chambers (TK Systems Co., Ltd., Asan, Korea) in which the thermoneutral birds were kept at 29.0 °C ± 1 and those for HS at 38.0 °C ± 1 for 3 h. The body weight of individual birds (all 12 birds per treatment) was measured before and after the heat exposure. Feed intake of each replicate was calculated by subtracting the feed refusal from the total amount of feed given at the beginning of the trial. Directly after the heat challenge, randomly selected individuals from each treatment were selected for blood and tissue sampling.

### 2.3. Blood, and Tissue Collection

In Experiment 1, six birds from each treatment were randomly selected and euthanized using carbon dioxide for blood collection. Blood was collected from heart puncture using sterilized syringes and was transferred into heparinized vacuum containers (#367874, BD Co., Ltd., Franklin Lakes, NJ, USA). Blood samples were then centrifuged immediately at 2000× *g* for 10 min at 4 °C. The plasma was collected and stored at −20 °C for later analysis. 

In Experiment 2, six birds from each treatment were randomly selected for blood and tissue collection. Euthanasia and blood collection were performed in the same way as explained in Experiment 1. Tissues (liver, spleen, bursa, heart, proventriculus, and gizzard) were collected, measured, weighed, and immediately snap-frozen in liquid nitrogen and stored at −80 °C for later analysis.

### 2.4. Plasma Concentrations of Glucose, Metabolites, Electrolytes, and Oxidative Stress Markers

Plasma concentrations of glucose, metabolites, and electrolytes were measured using a VetTest Chemistry Analyzer (IDEXX Co., Ltd., Westbrook, ME, USA) with a dry-slide technology following the manufacturer guide. Free radical scavenging activity was determined using a 2,2-diphenyl-1-picrylhydrazyl radical scavenging activity assay (DPPH-RSA) based on the previous method described by Gerasopoulos et al. [16]. Briefly, 20 μL of plasma were added to 480 μL of 10 mmol/L sodium-potassium phosphate (pH 7.4), subsequently followed by the addition of 500 μL of 0.1 mmol/L of (DPPH) free radical, and the samples were incubated in the dark for 30 min at room temperature. The samples were centrifuged at 20,000× *g* for 3 min, and the absorbance was read at 520 nm. The percentage of inhibition was calculated based on the following formula: % inhibition = [1 − (A1/A0)] × 100, where A0 is the absorbance of the control, and A1 is the absorbance of test samples.

For MDA concentrations, the method described by Jyothi et al. [17] was used with slight modifications. Briefly, 500 μL of plasma was added to 500 μL of 40% trichloroacetic acid (TCA), followed by the addition of 1 mL of 0.67% thiobarbituric acid (TBA). The solution was then kept immediately for 45 min in a boiling water bath. Next, it was cooled in an ice-cold water bath for 5 min. After cooling, the solution was centrifuged at 9950× *g* for 30 s, and the absorbance of the supernatant was read at 530 nm.

### 2.5. Real-Time PCR for mRNA Quantification

RNA was extracted from 50 mg of liver tissues using the Trizo reagent (Thermo Fisher Scientific, Waltham, MA, USA) following the manufacturer’s guide. The optical density of each sample was then read at 260 and 280 nm using a Nanodrop (Thermo Scientific, Waltham, MA, USA) to determine their concentrations and purities. Afterward, cDNA was synthesized using a PrimeScript first-strand cDNA synthesis kit (Takara, Tokyo, Japan) following the manufacturer’s instructions. The cDNAs obtained were used for amplification of different genes using a real-time polymerase chain reaction.

Real-time PCR was realized using a StepOnePlu real-time PCR system (Life Technologies, Carlsbad, CA, USA). Each reaction well included 20 μL Power SYBR^TM^ green PCR master mix (Life Technologies, Carlsbad, CA, USA), and 10 pmol of forward and reverse primers specific for each gene and cDNA. Table 1 is presented for primer sequence information related to genes, and GAPDH was used as a housekeeping gene. Cycling conditions of real-time PCR for primer annealing and subsequent melting curve analysis were: 10 min at 95 °C then followed by 40 cycles of 15 s at 95 °C and 1 min at 60 °C. To estimate the change in gene expression for RNA in different samples, the 2^−ΔΔCt^ method against the mean value of G0-TN was used as a control.

### 2.6. Statistical Analysis

The data in Experiment 1 were analyzed by one-way ANOVA followed by a Tukey’s post-hoc test. The level of statistical significance was set at *p* < 0.05. Dose-related effects of GABA were analyzed using polynomial regression analysis in the absence of the CON group. In Experiment 2, the data were analyzed by a two-way ANOVA procedure, in which the main effects were GABA concentration and temperature. A Tukey’s post-hoc test was performed to assess differences among means when an interaction was found. Differences among means were considered significant at *p* < 0.05 unless otherwise stated. Results are expressed as mean ± SEM. All the analyses were conducted using SPSS, version 25.0 (IBM SPSS Inc., Chicago, IL, USA).

## 3. Results

An in ovo injection of GABA did not seem to affect hatchability compared with the control group (Table 2). Although hatchlings’ body weights of G05 and G20 were not significantly different from CON or DDW, there was a significant difference between G05 and G20 (*p* < 0.05). A higher chick weight to egg weight ratio (CWEWR) was found in chicks at G05 compared with CON (*p* < 0.05) (Table 2). There were significant linear and quadratic effects in the chicks’ body weight at hatch and CWEWR with increasing in ovo GABA doses (*p* < 0.01).

Aspartate aminotransferase (AST) in plasma was significantly higher in G05 in comparison with CON (*p* < 0.05) (Table 3).

On the other hand, cholesterol concentrations were significantly decreased by the in ovo GABA administration (G05 and G10) compared with CON (*p* < 0.05). Moreover, there was a significant quadratic effect in blood cholesterol content with an increase in GABA injection doses (*p* < 0.05). Overall, the in ovo GABA administrations tended to increase AST while decreasing cholesterol (Table 3). In the plasma of hatchlings, DPPH-RSA was significantly increased (*p* < 0.01) in G10 compared with CON, but MDA was not significantly changed by the in ovo GABA treatments (Table 4).

Both the in ovo injection of GABA and heat exposure did not affect body weight, feed intake for 3 h (Table 5), or relative organ weights (Table 6). There were no significant interactions in any of these parameters.

Table 7 shows the effects of short-term HS, in ovo GABA injection, and their interactions on oxidative stress biomarkers in the plasma of ten-day-old chicks. GABA significantly increased DPPH-RSA (*p* = 0.01) but decreased MDA (*p* < 0.05). HS did not affect DPPH-RSA but significantly increased MDA (*p* < 0.05). A significant interaction was detected between GABA and temperature (*p* < 0.05) for MDA. 

**Figure 1:**
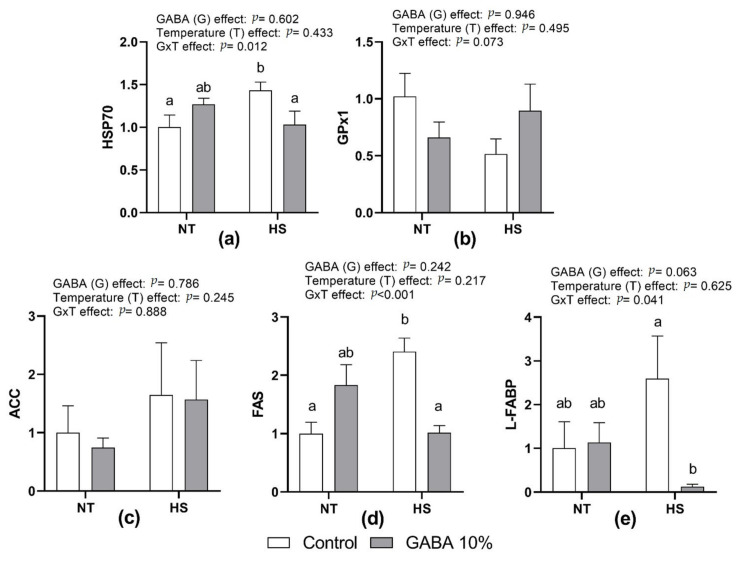
Effects of in ovo injection of GABA on relative mRNA expression of hepatic HSP70 (**a**), GPx1 (**b**), ACC (**c**), FAS (**d**), and L-FABP (**e**) in 10-day-old chicks exposed to acute heat stress for 3 h. At 17.5 days of incubation, eggs were in ovo injected with 0.6 mL of 10% GABA or not. Chicks obtained from each treatment were grown for 10 days, and were then divided into two subgroups. They were subsequently submitted to either heat stress (HS) at 38 ± 1 °C or thermoneutral temperature (NT) at 29 ± 1°C for 3 h. Data show mean ± SEM (n = 6). a,b: different letters indicate significant differences (*p* < 0.05) within all groups. Abbreviations: HSP70, heat-shock protein 70; GPx1, glutathione peroxidase 1; ACC: acetyl Co-A carboxylase; FAS, fatty acid synthetase; and L-FABP, liver fatty acid-binding protein.

Figure 1 presents the effect of HS, in ovo injection of GABA, and their interactions on the relative expression of HSP70, GPx1, ACC, FAS, and L-FABP mRNA in the liver. There were no significant differences in HSP70, FAS, and L-FABP between G0 and G10 at NT. However, in ovo GABA significantly reduced HSP70, FAS, and L-FABP in HS chicks, resulting in significant interactions (*p* < 0.05, *p* < 0.001, and *p* < 0.05, respectively). There were no significant effects of treatments and interaction on GPx1 and ACC expression.

## 4. Discussion

GABA is a four-carbon non-protein amino acid distributed widely among plants and animals [19]. An in ovo injection of various supplements including amino acids was extensively studied in the last decade [20,21]. In fact, amino acids, when injected in ovo during the first week of incubation [22] or at the end of embryonic life [23], improved or failed to trigger hatchability reduction. This can be explained by the fact that amino acids are involved in embryonic energy utilization during incubation and especially during the last incubation days [24]. In this study, an in ovo injection of GABA at 17.5 days of incubation did not reduce hatchability even though its concentrations varied widely from 5% to 20%. This result indicates that GABA is not detrimental to embryonic growth and might help facilitate the hatching process.

To our knowledge, this study is the first to evaluate the effects of in ovo GABA injections; however, dietary GABA supplementation has been extensively studied in chickens. A study found that supplementation of GABA under normal temperatures could improve body weight in broilers [10]. Similar results showed that GABA supplementation resulted in body weight gain in Ross broilers under the thermoneutral condition [11]. In our study, an in ovo GABA injection improved hatchling weight, and the heaviest came with the 5% GABA treatment. As a result, CWEWR was higher in chicks of the same treatment. This effect may be related to GABA’s influence on lipid metabolism [11]. Therefore, an in ovo injection of GABA might have resulted in better yolk utilization during the late embryo stage. Previous reports mentioned that there is a positive correlation between chick weight at hatch and broiler final weight [25]. Thus, an in ovo injection of GABA might help improve broiler weight at the slaughtering age.

In ovo fed nutrients are stored as reserves and used during hatching until the end of the first week after hatch [26]. Moreover, it appears that the effects of in ovo feeding were consistently noticed within the first two weeks of the chicks’ life [27]. Therefore, this study attempted to evaluate the thermal resistance of broiler chicks at ten days of age. Plasma concentrations of glucose, metabolites, and electrolytes appear to be a crucial tool for diagnostics, as they can be used to indicate metabolism and disease [28]. An in ovo injection of GABA increased plasma AST concentrations. Similar effects were found when GABA was supplemented in broilers [11]. Cholesterol is an essential component of animal cells and is found in substantial concentrations in plasma [29]. In this study, an in ovo injection of GABA at 5% and 10% significantly decreased total cholesterol in plasma. Comparable results, along with lipoprotein reduction, were observed in previous studies when GABA was supplemented to laying hens [30].

No GABA and temperature effects were detected on body weight and feed intake during the challenge, consistent with the results of Wickramasuriya et al. [31] that acute HS in 14-day-old chicks did not affect growth parameters. These results might be explained by the fact that growth parameters are generally not affected during short-term heat exposure. Moreover, ten-day-old chicks are relatively more resistant to heat exposure than those in marketable broilers.

Plasma MDA levels are closely related to the degree of cell damage that occurs during lipid oxidation [32]. DPPH-RSA is a common method used to assess TAC in plasma [33]. In our study, an in ovo injection of GABA resulted in enhanced TAC in broilers at hatch and at ten days of age under short-term heat exposure. Similarly, GABA supplementation increased TAC in chickens under HS [34], indicating that an in ovo GABA injection could instigate the retrieval of antioxidant functions after HS exposure. In broilers, HS has been recognized as an inducer to increase MDA levels [35]. An in ovo injection of 10% GABA brought back plasma MDA levels in HS chickens to values similar to those in thermoneutral conditions. In broilers, GABA supplementations had comparable effects on MDA concentrations [10]. GABA’s effects on reducing oxidation levels and improving TAC may be related to its ability to promote glutamate levels [36], thus enhancing antioxidant enzymes such as glutathione peroxidase activity [10,34]. In poultry, glutathione peroxidase is one of the main enzymes responsible for the first line of antioxidant defence [37]. Moreover, glutathione peroxidase plays a role in the detoxification of hydrogen peroxide, organic hydroperoxides, and lipid peroxide, thus enhancing the protection of cell membrane structure and functions [38]. Increased concentrations of glutathione peroxidase have been reported in blood or organs when GABA was supplemented under HS [10,34]. In the current study, however, GPx1 mRNA levels were not significantly affected. The higher resistance and shorter exposure of young chicks to heat may account for the different results obtained.

HSPs are well known for their roles in stabilizing the internal environment as well as improving the survival of stressed cells [39]. Heat exposure of broilers for a specific period resulted in upregulation of hepatic HSP70 gene expression [40,41]. As expected in our study, mRNA levels of HSP70 were significantly increased after 3h of HS. Interestingly, the present study also revealed that an in ovo injection of GABA could downregulate hepatic HSP70 gene expression in HS broilers, suggesting potential effects of GABA supplementation on reducing mRNA levels of HSP70 during HS. Previous reports demonstrated a strong correlation between HSP70 gene expression and thermotolerance in poultry [40]. Therefore, these results could be attributed to GABA’s role in decreasing the deleterious effects of HS in broilers.

The effects of high ambient temperatures on fatty acid metabolism are well documented in chickens [42]. Indeed, HS induces a reduction of a broiler’s physical activity and a decline in their basal metabolic rate, thus sparing energy stored as fat [43]. Since an in ovo injection of GABA reduced plasma lipid peroxidation levels in heat-exposed chicks, we evaluated the outcome of HS and an in ovo injection of GABA on hepatic expression of key genes involved in regulating the lipid metabolic pathway in broilers. ACC is involved in the conversion of acetyl-CoA into malonyl-CoA and then into palmitate [44]. Previous reports suggested that L-FABP plays a physiological role in hepatic lipid disposal as well as metabolic utilization of fatty acids [45]. FAS determines the maximum capacity for fatty acid synthesis in tissues [46]. Our findings showed that HS upregulates the relative expression of L-FABP and FAS in control birds. However, an in ovo injection of 10% GABA downregulated the expression of the same genes during heat challenge. Indeed, FAS mRNA expression was upregulated in Ross broilers but downregulated in Cobb broilers by GABA supplementation, indicating that the expression of FAS is strain-specific [11]. Unfortunately, the mechanism by which GABA could regulate fatty acid metabolism remains unclear. Indeed, it is possible that GABA might activate the adenosine monophosphate-activated protein kinase (AMPK) pathway for regulating fatty acid metabolism [11]. However, evidence shows that fat synthesis in chickens is under the control of the liver X receptor α (LXRα) pathway [47]. Therefore, further research is needed to address the mechanism by which GABA could induce a reduction in hepatic fat synthesis.

## 5. Conclusions

In conclusion, an in ovo injection of 5% GABA may improve post-hatch performance due to increased CWEWR at hatch. However, a decrease in plasma cholesterol and MDA, an increase in total antioxidant capacity, and a lower expression of hepatic FAS, L-FABP, and HSP70 mRNA may be indicators of heat tolerance. An in ovo injection of 10% GABA was found to help chickens have better heat resistance by regulating these parameters when exposed to HS. More research is needed to elucidate the potential effects of an in ovo injection of GABA in marketable broilers.

## Figures and Tables

**Table 1:** Primer sequences used to evaluate gene expression in the liver of 10-day-old broilers.

Gene	Sequence	Accession Number	Reference
ACC	F: CACTTCGAGGCGAAAAAC	XM_015295697.2	This study
	R: GGAGCAAATCCATGACCA		
FAS	F: CAATGGACTTCATGCCTC	NM_205155.3	This study
	R: GCTGGGTACTGGAAGACA		
GAPDH	F: TTGGCATTGTGGAGGGTCTTA	NM_204305.1	This study
	R: GTGGACGCTGGGATGATGTT		
GPx1	F: AACCAATTCGGGCACCAG	NM_001277853.2	[18]
	R: CCGTTCACCTCGCACTTCTC		
HSP70	F: GCTGAACAAGAGCATCAATCCA	AY143693.1	This study
	R: CAGGAGCAGATCTTGCACATTT		
L-FABP	F: GAAGGGTAAGGACATCAA	NM_204192.3	This study
	R: TCGGTCACGGATTTCAGC		

Abbreviations: ACC: Acetyl-CoA carboxylase; FAS: Fatty acid synthase; GAPDH: glyceraldehyde-3-phosphate dehydrogenase; GPx1: glutathione peroxidase 1; HSP70: heat shock protein 70; L-FABP: Liver type fatty acid-binding protein.

**Table 2:** Effects of in ovo administration of GABA on hatchability, chicks body weight at hatch, and chick weight to egg weight ratio.

Parameters	Treatments					*p*-Value		
	CON	DDW	G05	G10	G20	ANOVA ^1^	Lin ^2^	Quad ^2^
Egg numbers	42	42	42	42	42	NA	NA	NA
Egg weight (g)	61.0 ± 0.5	62.0 ± 0.3	61.0 ± 0.4	61.2 ± 0.5	60.8 ± 0.3	NA	NA	NA
Hatchability (%)	95.2	97.6	95.2	95.2	92.7	NA	NA	NA
Body weight at hatch (g)	40.9 ± 0.6 ^ab^	41.5 ± 0.5 ^ab^	42.2 ± 0.4 ^b^	41.1 ± 0.4 ^ab^	39.7 ± 0.5 ^a^	0.003	0.001	0.002
CWEWR (%)	66.5 ± 0.7 ^a^	67.9 ± 0.5 ^ab^	69.1 ± 0.5 ^b^	67.2 ± 0.7 ^ab^	65.7 ± 0.3 ^a^	0.013	0.001	0.002

^1^*p*-values of all treatment groups. ^2^*p*-values of all treatment groups except for the non-injected control group. At 17.5 days of incubation, eggs were in ovo injected with 0.6 mL of 0% (DDW), 5% (G05), 10% (G10), and 20% GABA (G20), or not (CON). Data show mean ± SEM (n = 12). a,b: different letters indicate significant differences (*p* < 0.05). Abbreviations: CWEWR: chick weight to egg weight ratio; NA: not applicable; Lin: linear effect; Quad: quadratic effect.

**Table 3:** Effects of in ovo injection of GABA on plasma concentrations of glucose, metabolites, and electrolytes of hatchlings.

Parameters	Treatments					*p*-Value		
	CON	DDW	G05	G10	G20	ANOVA ^1^	Lin ^2^	Quad ^2^
Glucose (mg/dL)	250.3 ± 14.5	248.5 ± 15.4	243.7 ± 13.8	231.3 ± 7.0	226.7 ± 9.6	0.591	0.331	0.52
Ammonia (umol/L)	405.6 ± 57.3	524.8 ± 14.0	510.8 ± 71.0	428.6 ± 35.7	396 ± 76.4	0.327	0.064	0.183
Albumin (g/dL)	0.9 ± 0.08	1.1 ± 0.08	1 ± 0.1	0.9 ± 0.06	0.9 ± 0.1	0.457	0.191	0.317
Total protein (g/dL)	3 ± 0.2	3.5 ± 0.2	3.2 ± 0.2	2.6 ± 0.2	3.1 ± 0.3	0.202	0.473	0.109
Phosphorus (mg/dL)	4.5 ± 0.2	4.4 ± 0.2	4 ± 0.2	4.1 ± 0.1	4.3 ± 0.2	0.418	0.936	0.641
Calcium (mg/dL)	9 ± 0.1	9.3 ± 0.4	9.2 ± 0.2	8.8 ± 0.2	9.1 ± 0.4	0.805	0.987	0.419
Uric acid (mg/dL)	8.9 ± 2.5	4.9 ± 0.9	7.4 ± 0.6	5.7 ± 0.6	7.5 ± 0.8	0.248	0.69	0.858
Aspartate aminotransferase (U/L)	199.5 ± 23.4 ^a^	295.7 ± 46.5 ^ab^	389.3 ± 50.1 ^b^	305.8 ± 51.4 ^ab^	333.8 ± 34.5 ^ab^	0.048	0.597	0.412
Triglicerides (mg/dL)	63.5 ± 8.9	64.2 ± 4.3	55.7 ± 5.9	75.5 ± 10.8	63.3 ± 9.5	0.574	0.651	0.845
Cholesterol (mg/dL)	442.8 ± 23.5 ^a^	418.8 ± 14.1 ^bc^	361 ± 12.5 ^ab^	345.2 ± 15.0 ^ac^	384.2 ± 20.7 ^abc^	0.003	0.151	0.037
Amylase (U/L)	1040.2 ± 79.0	1084.5 ± 77.5	1133.8 ± 93.1	1037.2 ± 71.8	1056.7 ± 127.1	0.941	0.338	0.531

^1^ *p*-values of all treatment groups. ^2^*p*-values of all treatment groups except for the non-injected control group. At 17.5 days of incubation, eggs were in ovo injected with 0.6 mL of 0% (DDW), 5% (G05), 10% (G10), and 20% GABA (G20), or not (CON). Chicks obtained from each treatment were sampled. Data show mean ± SEM (n = 6). ^a–c^: different letters indicate significant differences (*p* < 0.05). Abbreviations: Lin: linear effect; Quad: quadratic effect.

**Table 4:** Effects of in ovo injection of GABA on oxidative stress markers in blood biochemicals of hatchlings.

Parameters	Treatments					*p*-Value		
	CON	DDW	G05	G10	G20	ANOVA ^1^	Lin ^2^	Quad ^2^
DPPH-RSA (%)	27.81 ± 3.4 ^a^	39.85 ± 3.6 ^ab^	37.15 ± 4.9 ^ab^	47.51 ± 0.6 ^b^	39.85 ± 2.5 ^ab^	0.007	0.608	0.365
MDA (nmol/mL)	0.66 ± 0.1	1.21 ± 0.3	0.98 ± 0.5	0.96 ± 0.5	0.84 ± 0.2	0.329	0.137	0.252

^1^ *p*-values of all treatment groups. ^2^*p*-values of all treatment groups except for the non-injected control group. At 17.5 days of incubation, eggs were in ovo injected with 0.6 mL of 0% (DDW), 5% (G05), 10% (G10), and 20% GABA (G20), or not (CON). Chicks obtained from each treatment were sampled. Data show mean ± SEM (n = 6). ^a,b^: different letters indicate significant differences (*p* < 0.05). Abbreviations: Lin: linear effect; Quad: quadratic effect.

**Table 5:** Effect of in ovo injection of GABA on body weight variation of 10-day-old chicks exposed to acute heat stress for 3 h.

Treatments		Body Weight (g)		Feed Intake (g)
GABA	Temperature	Before	After	
0	Normal	236 ± 5.7	235.7 ± 7.4	9.5 ± 1.4
	Heat-stress	232.2 ± 3.4	249.3 ± 7.4	12 ± 1.3
10	Normal	226. ± 15.2	226.7 ± 15.7	8.8 ± 1.5
	Heat-stress	235.5 ± 19.3	231.2 ± 21.8	9.8 ± 1.4
Main effects				
GABA	0	234.1 ± 3.2	242.5 ± 5.5	10.7 ± 1.5
	10	230.8 ± 11.5	229 ± 12.5	9.3 ± 1.4
Temperature	Normal	231 ± 7	231.2 ± 8.2	9.1 ± 1.4
	Heat-stress	233.8 ± 9.1	240.2 ± 11.2	10.9 ± 1.4
*p* value				
GABA		0.797	0.367	0.157
Temperature		0.829	0.543	0.100
GABA*Temperature		0.611	0.760	0.451

At 17.5 days of incubation, eggs were in ovo injected with 0.6 mL of 10% GABA or not. Chicks obtained from each treatment were grown for 10 days and were then divided into two subgroups. They were subsequently submitted to either heat stress (HS) at 38 ± 1 °C or thermoneutral temperature (NT) at 29 ± 1 °C for 3 h. Data show mean ± SEM (n = 12).

**Table 6:** Effect of in ovo injection of GABA on relative organ weight (g/100g BW) of 10-day-old chicks exposed to acute heat stress for 3 h.

Treatments		Liver	Gizzard	Proventriculus	Heart	Bursa	Spleen
GABA	Temperature						
0	Normal	3.85 ± 0.3	2.55 ± 0.2	0.88 ± 0.0	0.80 ± 0.0	0.14 ± 0.0	0.09 ± 0.0
	Heat-stress	3.22 ± 0.3	2.45 ± 0.2	0.82 ± 0.0	0.80 ± 0.0	0.09 ± 0.0	0.12 ± 0.0
10	Normal	3.73 ± 0.3	2.25 ± 0.2	0.84 ± 0.0	0.77 ± 0.0	0.12 ± 0.0	0.11 ± 0.0
	Heat-stress	3.67 ± 0.3	2.58 ± 0.2	0.85 ± 0.0	0.81 ± 0.0	0.14 ± 0.0	0.09 ± 0.0
Main effects							
GABA	0	3.54 ± 0.2	2.50 ± 0.1	0.85 ± 0.0	0.85 ± 0.0	0.11 ± 0.0	0.11 ± 0.0
	10	3.70 ± 0.2	2.42 ± 0.1	0.85 ± 0.0	0.85 ± 0.0	0.13 ± 0.0	0.10 ± 0.0
Temperature	Normal	3.79 ± 0.2	2.40 ± 0.1	0.86 ± 0.0	0.78 ± 0.0	0.13 ± 0.0	0.10 ± 0.0
	Heat-stress	3.44 ± 0.2	2.52 ± 0.1	0.84 ± 0.0	0.81 ± 0.0	0.11 ± 0.0	0.10 ± 0.0
*p* value							
GABA		0.584	0.604	0.844	0.845	0.251	0.75
Temperature		0.245	0.457	0.449	0.545	0.225	0.861
GABA*Temperature		0.339	0.173	0.379	0.629	0.016	0.157

At 17.5 days of incubation, eggs were in ovo injected with 0.6 mL of 10% GABA or not. Chicks obtained from each treat-ment were grown for 10 days, and were then divided into two subgroups. They were subsequently submitted to either heat stress (HS) at 38 ± 1 °C or thermoneutral temperature (NT) at 29± 1 °C for 3 h. Data show mean ± SEM (n = 6).

**Table 7:** Effect of in ovo injection of GABA on plasma oxidative stress markers of 10-day-old chicks exposed to acute heat stress for 3 h.

Treatments		DPPH-RSA (%)	MDA (nmol/dL)
GABA	Temperature		
0	Normal	54.8 ± 1.2	2.7 ± 0.2 ^a^
	Heat-stress	54.4 ± 2.7	6.4 ± 1.5 ^b^
10	Normal	57.9 ± 0.7	2.6 ± 0.2 ^a^
	Heat-stress	60.1 ± 0.4	2.5 ± 0.4 ^a^
Main effects			
GABA	0	54.6 ± 1.4	4.6 ± 0.9
	10	59.0 ± 0.5	2.5 ± 0.2
Temperature	Normal	56.4 ± 0.8	2.7 ± 0.1
	Heat-stress	57.3 ± 1.5	4.5 ± 0.9
*p* value			
GABA		0.010	0.018
Temperature		0.568	0.034
GABA*Temperature		0.408	0.029

At 17.5 days of incubation, eggs were in ovo injected with 0.6 mL of 10% GABA or not. Chicks obtained from each treatment were grown for 10 days, and were then divided into two subgroups. They were subsequently submitted to either heat stress (HS) at 38 ± 1 °C or thermoneutral temperature (NT) at 29 ± 1 °C for 3 h. Data show mean ± SEM (n = 6). ^a,b^: different letters indicate significant differences (*p* < 0.05). Abbreviations: DPPH-RSA: 2, 2-diphenyl-1-picrylhydrazyl radical scavenging activity; MDA: malondialdehyde.

## Data Availability

All the relevant data is available in the paper.
